# Health Through a Human Right Lens at the US-Mexico Border: Increasing Access to Healthcare for Central American Immigrants

**DOI:** 10.3389/fpubh.2022.858402

**Published:** 2022-07-12

**Authors:** Carolina Venturi, Diana Guízar-Sánchez, María Elena Ramos-Tovar, Melissa Torres, Flor D. Avellaneda, Luis R. Torres-Hostos, Omar Matuk-Villazon

**Affiliations:** ^1^College of Medicine, University of Houston, Houston, OH, United States; ^2^Physiology Department, Faculty of Medicine, Universidad Nacional Autónoma de México, México City, Mexico; ^3^Sistema Nacional de Investigadores, Nivel SNI II, Facultad de Trabajo Social y Desarrollo Humano, Universidad Autónoma de Nuevo León, San Nicolás de los Garza, Mexico; ^4^Baylor College of Medicine, Houston, TX, United States; ^5^School of Social Work, Baylor University, Waco, TX, United States; ^6^School of Social Work, The University of Texas Rio Grande Valley, Edinburg, TX, United States

**Keywords:** human rights, immigrants, social inequalities, Northern Triangle of Central America, binational health insurance

## Abstract

The number of immigrants seeking entry into the U.S. through asylum requests or through irregular means is increasing, and most come from the Northern Triangle of El Salvador, Guatemala, and Honduras. Immigrants come fleeing extreme poverty, violence, health and social inequities, and drastic climate changes. Most had limited access to healthcare at home, and even more limited care along the journey. Those that are allowed entry into the U.S., are confronted with feeling unwelcome in many communities, having to navigate an array of local, state, and federal laws that regulate access to healthcare. We need immigration policies that preserve the health, dignity with a multinational policy for provision of healthcare through a human rights lens from point of origin to point of destination.

## Introduction

External migration is an old phenomenon. Historically and presently, migration surges in response to decreased economic opportunities in one country and increased economic opportunities in another; searching for a higher quality of life; natural or human-made disasters; or escaping political oppression and social turmoil (1–4). Although the causes for migration may remain unchanged, the composition of populations migrating to the United States of America has changed repeatedly. Current waves of immigrants are increasingly coming from the Northern Triangle countries of Central America (Guatemala, Honduras, and El Salvador) (5, 6).

According to the U.S. Customs and Border Protection (CBP), the number of encounters with immigrants along the U.S-Mexico border rose from 977,509 in the 2019 fiscal year (FY) to 1,734,686 in fiscal year 2021 (7). Most of these encounters occurred at the southwest border (97%), and of these encounters, “63% involved people from countries other than Mexico and most were immigrants of the northern triangle” (8). Unlike pre-COVID encounters, which resulted in detentions, most post-COVID contacts resulted in expulsions. Fear of interacting with government institutions, together with the current risk of immediate deportation, could potentially lead to delays in seeking medical attention, even when there is an urgent need for it.

Facing dire socioeconomic inequities, violence, political instability, environmental crises and drastic climate changes in their countries of origin, Northern Triangle expatriates migrate with characteristics that further exacerbate their risks for poor health and mental health outcomes when in transit and at their place of destination (4, 9, 10). There is ample research support for the relationship between social determinants of health (SDH) and the health of those exposed to such circumstances. The World Health Organization lists the social and economic environment, the physical environment, and the person's individual characteristics and behaviors as the common determinants of health (11). In this regard, and among other risk factors, most Guatemalans, Salvadorans, and Hondurans lack a high school diploma, lack English proficiency, earn the lowest median household income for Central Americans, and work in the service industry; all factors that perpetuate the heightened risk to experience inequity (12, 13). In tandem with these negative social determinants of health, the discrimination and ostracization Guatemalan, Salvadoran, and Honduran immigrants face further identifies them as vulnerable, marginalized populations which can impact their health and even aggravate pre-existing conditions.

In an attempt to protect these vulnerable populations, migrant human rights protection agreements abound. The International Organization for Migration (IOM), the Pan American Health Organization (PAHO), the World Health Organization (WHO), and many other national and international agencies have agreements with participating member nations that highlight the urgency and immediacy of protecting immigrants' human rights, which include access to health care without regard for migration status (5, 14). Yet, the policies and systems in place continue to fail immigrants. Cooperation between countries of origin, transit, and destination should be strengthened to address the causes, challenges, and conditions that aggravate rather than alleviate the negative risk factors and subsequent health outcomes.

## Policy Options and Implications: Health Inequities and Human Rights Violations

### Pre-migration

Health inequities, as defined by the WHO, “are differences in health status or in the distribution of health resources between different population groups, arising from the social conditions in which people are born, grow, live, work and age” (15). It is well-documented that those who reside within the Northern Triangle of El Salvador, Guatemala, and Honduras experience less opportunity for human development and high rates of poverty, violent crime, and low educational attainment. Thus, the determinants of health Central Americans in the Northern Triangle face reveal a discouraging panorama.

The Pew Research Center estimates that 50% of adult immigrants (25 years of age and above) from each country in the Northern Triangle have not completed a high school degree. This is a stark contrast to only 29% of all immigrants lacking a high school degree (16). Compounding the low educational attainment are financial burdens. According to the World Bank Organization, as of 2020 the economic output per person in Guatemala is 4,603.30 USD; El Salvador's per person output is 3,798.60 USD; and Honduras' per person output is 2,389.00 USD. Further, the portion of Northern Triangle people who have lived on 2 USD a day is high: 17% Hondurans, 10% Guatemalans, and 3% Salvadorans. Economically, the Northern Triangle is one of the most strained regions in the Western Hemisphere, and, when ranked by gross national product per capita, they are three of the bottom six Latin American countries. Additionally, the countries are plagued with high homicide rates—often ranking within the top five countries globally—and gang violence (17, 18). A quick look at these Northern Triangle countries' health care systems reveals their populations' resolve to thrive even in the face of further inequity.

#### El Salvador

El Salvador is one of 57 countries considered to have critical shortages of healthcare personnel. Current estimates reveal a physician density of 1.6 per 1,000 population, far below the WHO's suggested 23 physicians per 10,000 for sufficient primary healthcare coverage. The Ministry of Public Health manages its health care system, where district hospitals, in relation to national ones, are underfunded. The district hospitals also report a critical shortage of acute care nurses due to underfinancing. El Salvador's per capita health care spending is 450.00 USD (3, 4, 17, 19).

#### Guatemala

Guatemala's Ministry of Health intends to implement a basic primary care package of services to reduce inequities, where health services represent a financial burden not evenly distributed across the country. Further, an existent ethnic divide in the country perpetuates/accentuates disparities in access to health care services. Most nurses are trained at the auxiliary level, and perform tasks reserved for physicians due to shortages. Physician density is 0.93 per 1,000 population. Guatemala's per capita health care spending is 325.00 USD (3, 4, 17, 19).

#### Honduras

Honduras suffers a critical shortage for all healthcare personnel in all categories with a physician density of 0.37 per 1,000. The Ministry of Health is the major provider of healthcare, serving 88.3% of the population. Honduras' per capita expenditure is 263.00 USD (3, 4, 17, 19).

In addition to lack of adequate healthcare providers, inequities exist rural and urban communities, and between populations of differing socioeconomic status (17). Many healthcare providers in the USA lack cultural/contextual/linguistic proficiency to work with Spanish/Indigenous-speaking patients, often resulting in unequal encounters that can be experienced discriminatory and lead to poor health outcomes (20, 21). Classism, racism, and discrimination based on Indigenous status are structural phenomenon that have many ways of manifesting themselves and have been present in Latin America for centuries. They are complex phenomena, rooted in Latin American societies and built from racial or ethnic hierarchization and inferiorization (22). Latin American societies developed from racist ideologies that glorified white-mestizo national identity and denied Black and Indigenous roots and their contributions to the history and culture of national states (23).

Diversity has become not something to celebrate, but in many cases an excuse to discriminate and deny access to rights and opportunities to both Indigenous and/or Black residents/citizens/people. As with other groups, skin tone, socioeconomic status, discrimination, and racism are directly associated in a society that hierarchizes differences, some of which are seen as a mark of inferiority and used to legitimize inequalities, to the point that prejudices and discriminations are created that end up being naturalized (24, 25). To date, research on racial discrimination and health has focused primarily on interpersonal discrimination as a psychosocial stressor, affecting psychological wellbeing, mental health, and related unhealthy practices (e.g., sleep disturbance, eating patterns, and the consumption of psychoactive substances, including cigarettes, alcohol, and drugs).

Furthermore, interpersonal racism can lead to various biomarkers of disease and wellbeing, including allostatic load, inflammatory markers, and hormonal dysregulation (26). Arceo-Gomez et al. (27) showed that allostatic load increased with age most notably among foreign-born Black persons of both sexes and among foreign-born Latina women.

### Transit

Whether to escape chaotic living conditions or to improve the quality of their lives, an increasingly higher number of Northern Triangle residents decide to migrate north to the United States of America. Most of them cite better job opportunities as their main reason, while others cite the desire to reunite with relatives (1–4). There are ~2,422 km between Guatemala's border and the Texas border, a journey which will traverse the states of Chiapas, Oaxaca, Veracruz, Tabasco, and Tamaulipas. Most undocumented immigrants from the Northern Triangle traverse Mexico on La Bestia. Operated by a group of private companies, La Bestia refers to cargo trains transporting different products to the United States. From the Guatemalan border, immigrants must reach the two closest train stations, which are in the states of Tabasco, Mexico, and Chiapas, Mexico. Dependent upon their place of destination, immigrants must choose one of the three routes: the gulf route for those who seek entry through Texas; the Pacific route for those who seek to enter through Arizona or California; and/or a central route for those who seek to enter through Western Texas or New Mexico. For immigrants unable to pay as much as 10,000.00 USD for smuggling fees, La Bestia is the only means of transportation. Yet, riding this cargo train only means paying less than what smuggling may cost, as migrants on board then face extortion and recruitment by gang members, extortion by unscrupulous law enforcement officials, sexual assault, and physical injury from unsafe travel conditions (28). The Tamaulipas Massacre exemplifies organized crime victimization of highly vulnerable migrants (28). In 2018, a total of 72 migrant men and women were shot dead allegedly at the hands of the Zetas Cartel. A survivor recounts how the Zetas attempted to extort them, forcefully recruit them, and believed the migrant caravan intended to work for the Gulf Cartel (their rivals).

Transiting through Mexico, immigrants often change train lines and stop at shelters run by civil society organizations, where they have access to healthcare services provided by several humanitarian organizations. Among them, Doctors Without Borders (DWB) provides medical and psychological care to immigrants and refugees along the migration routes through Mexico. DWB provides aid at shelters, hostels, hotels, and community kitchens. Traditionally, “Casas del Migrante” (migrant shelters) and local churches have provided the much-needed aid for immigrants from the start to the end of transit. Mostly supervised by clergy, these are autonomous organizations that maintain relationships with additional organizations, making them a relevant and extensive support network for immigrants. In cooperation with government institutions, NGOs, and international humanitarian-assistance agencies, these Casas del Migrante help mobilize resources toward the health of immigrants and can be important partners in a regional strategy to protect the health and human rights of immigrants in transit.

### Destination

Northern Triangle undocumented immigrants often cross at points along the Texas-Mexico border. The main reason for this lies in a policy that releases immigrants on their own recognizance when shelters exceed space-capacity, as is usually the case in the McAllen and Del Rio border stations (28, 29). In 2021, 97% of all apprehensions occurred at the southwest U.S.-Mexico border. The Texas- Mexico border area encompassing the Brownsville to El Paso region is a relevant point of entry, where an estimated 12% of all immigrants from the Northern Triangle countries reside, with the majority residing in Harris, Dallas, Tarrant, and Hidalgo counties (10, 30).

An estimated 100,000 Northern Triangle immigrants currently reside in Hidalgo County (located within the greater Rio Grande Valley, Texas). Unfortunately, the Rio Grande Valley's (RGV) existing infrastructure lacks the capacity to provide services or healthcare for this vulnerable population. The entire RGV region is designated as a medically underserved area, has a high percentage of individuals living below the federal poverty line, 41% percent of residents are not fluent in English, and health illiteracy is relatively high. Thus, opportunities and quality of life conditions for those immigrants who decide to reside in the RGV are further diminished.

Nevertheless, medical care for undocumented immigrants is available (in the RGV and United States at large) through Federally Qualified Health Centers (FHQC), which operate around the country. Funded by the Health Services and Research Administration and with a mandate to serve uninsured, underinsured, and indigent individuals, there are ~1,200 FQHCs in the U.S. and 72 in Texas. The RGV, a 4-county region the size of Connecticut and home to 1.5 million people, has three FQHCs: one in Brownsville, Texas (Brownsville Community Health Center); one in Harlingen, Texas (Su Clinica Familiar); and one in Alamo, Texas (Nuestra Clinica Del Valle) (31–34). As they provide health care services without regard to cost or immigration status, these centers represent an important safety net for those who have limited access to health care, and/or live-in rural areas.

Today, no public policy for binational or regional/multinational health insurance exists. Aside from FQHCs and other small, safety net clinics, Central American immigrants in the U.S. receive care from the U.S. health system if they can afford the services or are documented. Migratory status determines their ability to access public or private health services, as seen by the current roll-out of the COVID-19 vaccine. There are a few exceptions, such as programs implemented by the Mexican government with different activities and services for Mexican nationals residing in the U.S. Implemented through the Institute of Mexicans Abroad (IME), such programs include *Binational Health Week* and *Ventanillas de Salud*. However, these programs tend to focus on large cities, are citizenship-dependent (i.e., Salvadoran or Honduran nationals would not qualify) and none provide comprehensive prevention or care for chronic or acute health conditions.

## Conclusions and Actionable Recommendations

Multiple national and/or global agreements are in place to protect immigrant human rights. Considering health as a human right, quoting the WHO, “The enjoyment of the highest attainable standard of health is one of the fundamental rights of every human being without distinction of race, religion, political ideology, economic or social condition” (35). Healthcare is a core component of the right to health. Additionally, multiple organizations exist to assess migration flows and patterns and to assist countries partnering with the organizations to establish frameworks for sustainable commitment to the protection of human rights and health for immigrants. Human rights, including the right to health, are recognized in numerous international instruments and branches of international law, but there is no single comprehensive international instrument protecting the rights of all those who migrate (36).

Most notably, and through the instrumental body of the Human Rights Council, the Universal Declaration of Human Rights has championed the cause since 1948 (33, 34, 37). On September 19, 2016, at the UN Summit, member states came together around one document: the New York Declaration for Refugees and Migrants, which expresses the unanimous agreement of the 193 UN member states to promote and protect the human rights of immigrants and share responsibility on a global scale. Further, on December 10, 2018, through an intergovernmental conference on international migration held in Morocco, UN member states adopted the Global Compact for Safe, Orderly, and Regular Migration (GCM). It represents the first intergovernmental negotiated document, prepared under the auspices of the United Nations, to cover all dimensions of international migration in a holistic and comprehensive manner (14).

As can be seen, infrastructure which helps address the health equity and the human rights of migrants exists. However, the discretionary participation of member states results in eroded relevance and a failure to attain the goals these agreements were intended to achieve. The existence of these framework agreements and the time and effort put into them demand accountability. Understandably, this is a complex undertaking. But the protection of the most vulnerable communities among us should be a moral imperative. A failure to comply with these agreements should have consequences. And compliance should be paired with evaluation models that can assess and quantify their impact on the health and human rights of migrating communities.

Social, economic, political, and climate conditions in the Northern Triangle Central American countries thwart its inhabitants' birthright to pursue fundamental human needs. Many are oppressed, unable to provide safety or basic needs for themselves or their families, socially disenfranchised to the extent of statelessness. This truly is an intensely irrational, surreal reality that many Central Americans from El Salvador, Honduras, and Guatemala experience as they attempt to flee living conditions in their countries of origin. Unfortunately, it is an experience that remains with them while in transit and at destination. They undertake a harrowing journey north in search of a better life, and find themselves truly in a state of limbo, where borders' seem to disappear, they live in the shadows as they journey north, and their voices find no recipient and no echo. And those that are “lucky” enough to reach their destination are met with closed arms and increasing anti-immigration and anti-immigrant policies, especially in Texas.

We need more humane state and federal policies that ensure the rights and health of immigrants and refugees. We need expanded training opportunities for providers from all backgrounds to learn to identify and manage our own biases and stereotypes and increase our linguistic and cultural proficiency. Most professional licensing boards and state/federal agencies have “cultural competency training” requirements, but almost anything goes. Evidence-based cultural competency models and actual evidence of competency are not required. We must also provide more educational opportunities for low-income, U.S.- and foreign-born Hispanics/Latinos to join the provider ranks. They bring a deep, personal understanding of the challenges and strengths of their communities, and a commitment to improve health in these communities.

Observational studies have found that regions with a higher ratio of primary care physicians to specialists have better health outcomes, such as lower mortality, fewer emergency department visits, hospitalizations and procedures per capita, and lower costs. Reducing barriers to primary care, could improve the quality and cost of health care delivery in a country with a growing immigrant population; as has been attempted in several US states: (1) New York City has the largest public health care system in the country, comprising the Health and Hospitals Corporation (HHC) and the Community Health Care Association of New York State and immigrant health programs, which provide much of the health care for undocumented migrants, (2) California offers a Medi-Cal health insurance plan that provides a full range of low-cost health care options for uninsured Californians, with some benefits offered regardless of immigration status, (3) Houston, Texas, Access Care, a financial assistance program open to residents without insurance or documentation, is offered and provides access to discounted health care at more than 20 community clinics, a dental clinic, and surgical and other subspecialty clinics, (4) Massachusetts, all immigrants are entitled to some form of health coverage (38–40).

Finally, we must explore the development of a binational community of practice where physicians and other health and social services provides treating the same clients all along their journey can communicate and coordinate care. Language, legal, logistical, financial, and other barriers exist. However, these individuals who are migrating will continue to come in pursuit of a better life, better economic prospects, life, liberty, and the pursuit of justice. Coordination of care can lead to increased continuity of care, enhanced epidemiological surveillance, and better individual and population health outcomes (40). Training providers to better understand the needs of their immigrant patients is also required and reaching out to specific immigrant communities to educate about current laws and the system, especially education about health care rights.

## Author Contributions

OM-V, CV, DG-S, and LT-H: participating in the conception and design of the study. OM-V, CV, and DG-S: writing. All authors contributed to the discussion and revision of the manuscript for intellectual content. All authors contributed to the article and approved the submitted version.

## Conflict of Interest

The authors declare that the research was conducted in the absence of any commercial or financial relationships that could be construed as a potential conflict of interest.

## Publisher's Note

All claims expressed in this article are solely those of the authors and do not necessarily represent those of their affiliated organizations, or those of the publisher, the editors and the reviewers. Any product that may be evaluated in this article, or claim that may be made by its manufacturer, is not guaranteed or endorsed by the publisher.
